# Appendiceal intussusception due to endometriosis presenting as acute right lower quadrant pain

**DOI:** 10.1093/bjrcr/uaae032

**Published:** 2024-09-04

**Authors:** Ahmed O. El Sadaney, Ariel D Sandhu, Ahmad Parvinian, Avinash Nehra, Garima Suman

**Affiliations:** Department of Radiology, Mayo Clinic, Rochester, MN, 55905, United States; Department of Pathology, Mayo Clinic, Rochester, MN, 55905, United States; Department of Radiology, Mayo Clinic, Rochester, MN, 55905, United States; Department of Radiology, Mayo Clinic, Rochester, MN, 55905, United States; Department of Radiology, Mayo Clinic, Rochester, MN, 55905, United States

**Keywords:** appendix, endometriosis, intussusception, CT enterography

## Abstract

Appendiceal intussusception is a rare condition characterized by the telescoping or invagination of a portion or the entire appendix into the caecum or within the appendix itself. Diagnosing appendiceal intussusception can be challenging due to its rarity, non-specific symptoms, and lack of awareness among physicians. We present a case report of appendiceal intussusception caused by endometriosis presenting with recurrent abdominal pain in a young female that was initially missed on CT scan and laparoscopy and eventually diagnosed on CT enterography.

## Introduction

Appendiceal intussusception is a rare condition characterized by the telescoping or invagination of a portion or the entire appendix into the caecum or within the appendix itself. The reported incidence of appendiceal intussusception in surgical specimens is remarkably low, estimated at 0.01%.^1^ This condition tends to affect adults more than children, with adult females twice as likely to be affected compared to males. The clinical presentation is often non-specific and can be variable. The symptoms can include acute abdominal pain, features of bowel obstruction, bleeding per rectum, or can even be asymptomatic. We present a case report of appendiceal intussusception presenting with recurrent abdominal pain in a young female.

## Case presentation

**Figure 1.**
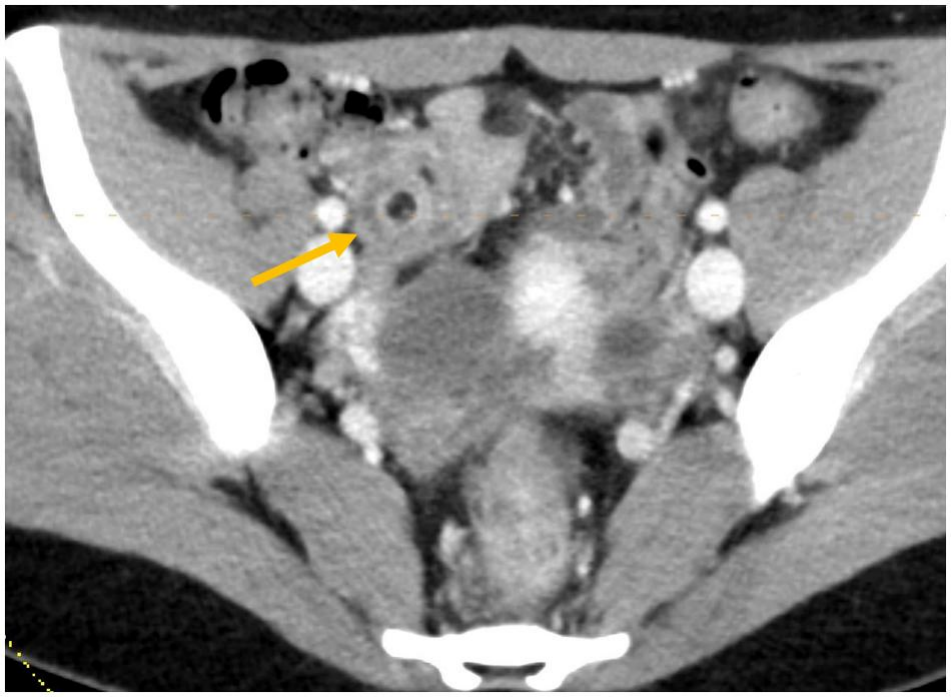
Axial CECT image through the right lower abdomen demonstrating target sign due to mesenteric fat telescoping into a bowel segment (arrow).

**Figure 2.**
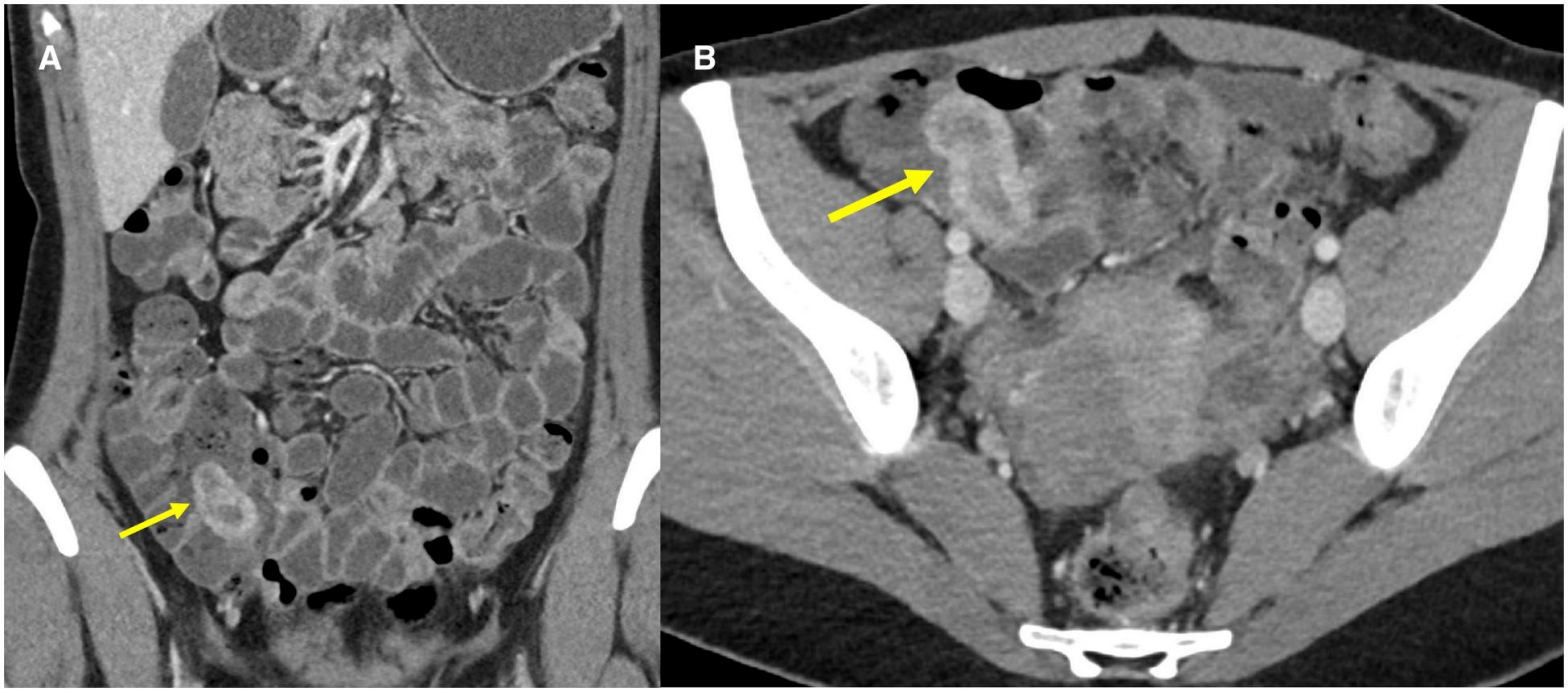
Coronal (A) and axial (B) CT enterography images demonstrate a blind-ending tubular structure with enhancing walls within the caecum (arrows).

A 21-year-old female presented to the emergency department with 12-h of sudden-onset right lower quadrant pain and nausea. The physical examination showed tenderness in her right lower quadrant without signs of rebound or guarding, and no tenderness at McBurney’s point or the umbilicus. Her laboratory tests were normal, except for mildly elevated leukocytes. An initial ultrasound of the abdomen and pelvis did not reveal significant abnormalities except for a 3 cm right ovarian cyst with low-level internal echoes, indicative of an endometrioma. A contrast-enhanced CT (CECT) of the abdomen and pelvis was subsequently performed. An axial CECT image demonstrated mesenteric fat telescoping into a bowel segment in the right lower quadrant, although the specific involved structures were not clearly identified (Figure 1). Due to persistent pain and indeterminate CT findings, she underwent a diagnostic laparoscopy, which was unrevealing. The patient was managed conservatively and discharged from the hospital upon symptomatic improvement. Over the next month, she experienced additional episodes of acute abdominal pain, leading to a follow-up CT enterography in an outpatient setting. Coronal and axial CT enterography images demonstrated a tubular structure within the caecum (Figure 2). The small bowel loops were normal. The tubular structure observed within the caecum was identified as the intussuscepted appendix. The ileocecal junction was distinctly visualized separate from the intussusception. The appendix was dilated along with mural thickening and hyperenhancement, suggestive of acute appendicitis. The CT scan did not reveal any discernible “lead point” associated with the intussusception.

## Discussion

The findings were consistent with complete appendiceal intussusception into the caecum with acute appendicitis. The differential diagnoses that can be considered on CT include inverted colonic diverticulum, caecal polyp, and ileocolic intussusception. However, the appendix should typically be seen separately in each of these conditions, unless a history of appendectomy is present.

**Figure 3.**
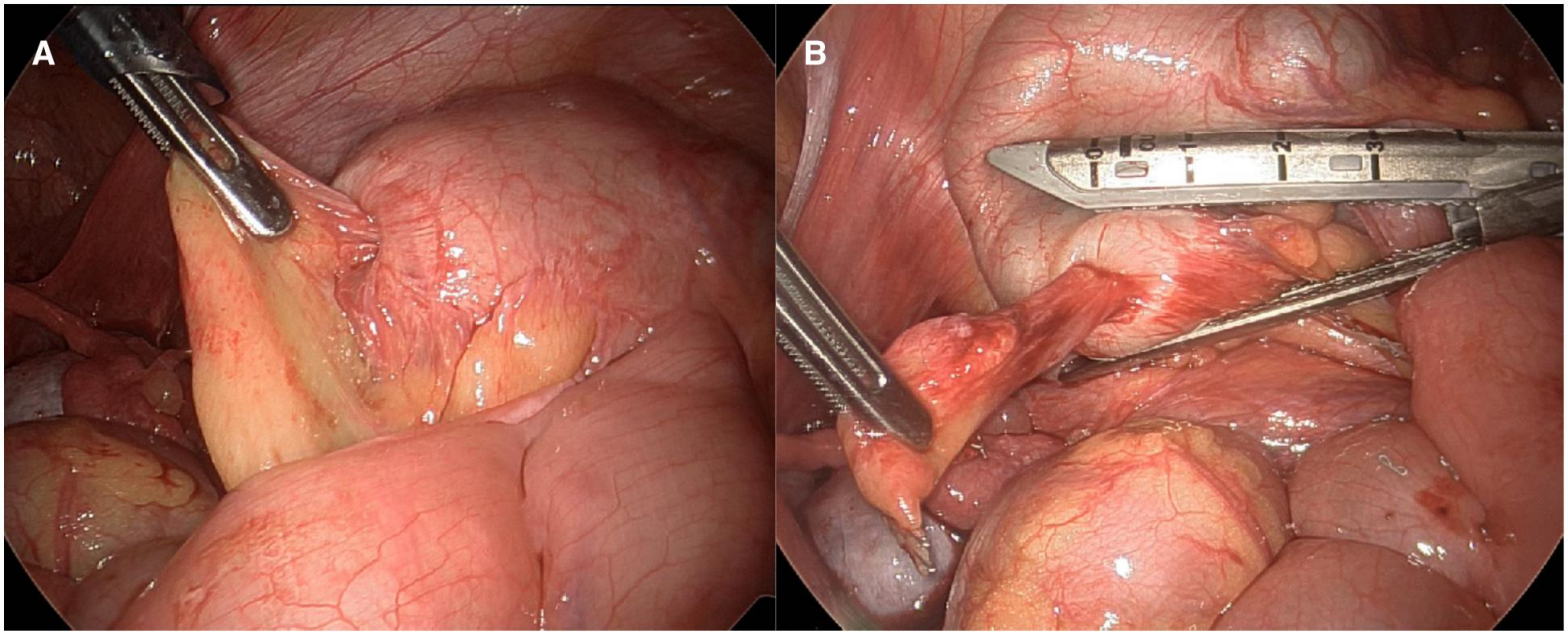
Intraoperative images (A and B) show partially reducible appendiceal intussusception.

**Figure 4.**
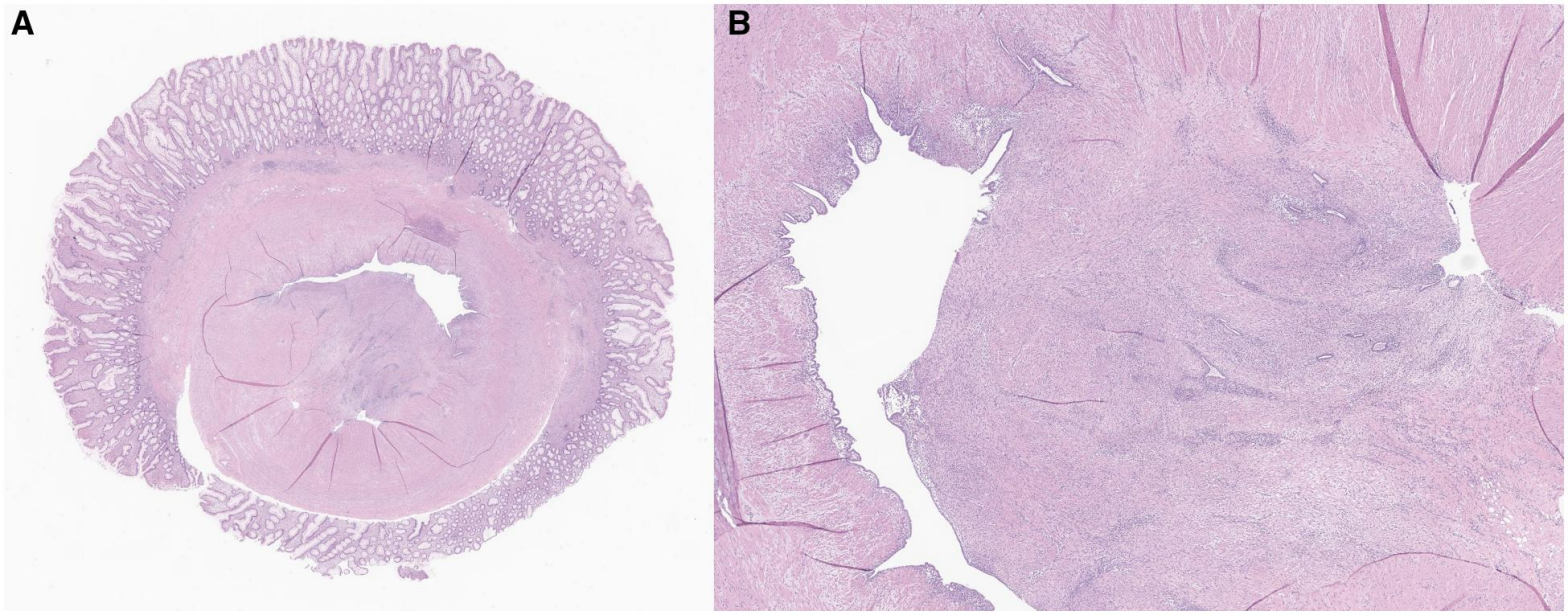
Low power H&E image (A) of a cross-section of the intussuscepted appendix, showing an inverted configuration of the layers of the appendiceal wall with the mucosa seen at the surface, and muscularis propria and serosa in the innermost layer. Higher power image (B) demonstrates near concentric involvement of the muscularis propria and serosal surface by endometriosis, resulting in tethering and adhesions.

Following the CT findings, the patient underwent an open appendectomy, confirming the diagnosis of appendiceal intussusception (Figure 3). The appendix could not be reduced intraoperatively, requiring a partial cecectomy for complete removal. The laparotomy also confirmed the presence of an endometrioma in the right ovary and showed numerous endometriotic deposits on the pelvic peritoneum. Histopathological examination revealed acute appendicitis with microscopic intramural endometriotic implants within the appendix (Figure 4).

Endometriosis is the most common lead point in adults, accounting for approximately 33% of cases, as in the current case. Other lead points include mucocele (19%), adenoma (11%), carcinoid (7%), and adenocarcinoma (6%).^2^ Additionally, there is a noted association with ulcerative colitis as another contributing condition.^3^ In paediatric population, appendiceal intussusception is frequently caused due to appendicitis, foreign body, and lymphoid hyperplasia, and endometriosis.

Diagnosing appendiceal intussusception is challenging, with literature suggesting that only 58% of cases are diagnosed presurgically.^2^ It may be missed on ultrasound due to artefacts from gas and faecal material in the caecum. When seen, ultrasound may demonstrate the concentric “target” or “donut” appearance of intussusception in the caecum, with concentric hyperechoic and hypoechoic layers of the bowel wall. Rarely, “lead points” such as endometrial implants may be seen on ultrasound as a hypoechoic nodule with posterior shadowing. Diagnosing appendiceal intussusception on CT can be challenging in cases with scant intraperitoneal fat and non-distended bowel loops. The appendix within the caecal lumen may be obscured by faecal material or may simply appear like caecal wall thickening. The typical CT appearance of appendiceal intussusception is a “sausage-shaped” blind-ending structure resembling an “inverted glove.” Associated telescoping of periappendiceal fat and ileocolic vessels may give a clue to the diagnosis. A lead point may be better demonstrated on CT in certain cases. Barium enema can demonstrate a “coiled-spring” appearance within the caecum from the contrast surrounding the unopacified appendix. However, it is rarely performed in adults for suspected intussusception. Endoscopy can be used but poses a risk of perforation or incomplete resection if biopsy or excision is attempted, as the appendix may resemble a polyp.^4^

McSwain’s classification categorizes appendiceal intussusception into 5 types based on the region of the appendix that undergoes intussusception^5^:

Type I: The tip of the appendix intussuscepts into the proximal appendix.
Type II: Any part of the appendix intussuscepts into its adjacent part.
Type III: The distal part of the appendix, at the junction with the caecum, intussuscepts into the caecum (not fully invaginated).
Type IV: Any part of the appendix intussuscepts backwards towards its distal part.
Type V: The entire appendix intussuscepts into the caecum.

## Conclusion

Our patient had McSwain Type V intussusception. The definitive management of appendiceal intussusception is appendectomy, often with caecal cuff resection.^6^ Appendiceal intussusception may be missed on laparoscopy if the appendix is completely invaginated. Our patient had a complete resolution of abdominal pain following the surgery.

## Learning points

- Diagnosing appendiceal intussusception can be challenging due to its non-specific presentation and low index of suspicion due to lack of awareness among clinicians.
- Endometriosis is the most common cause of appendiceal intussusception in adults.
- Definitive management requires open appendectomy with caecal cuff resection.

## Authors contributions

All authors participated in patient care relevant to the case report and take responsibility for the drafting, and critical revision of the manuscript for important intellectual content.
